# Minimal Extracorporeal Circulation and Microplegia in the Setting of Urgent Coronary Artery Bypass Grafting

**DOI:** 10.3390/jcm11247488

**Published:** 2022-12-17

**Authors:** Luca Koechlin, Brigitta Gahl, Jules Miazza, Urs Zenklusen, Bejtush Rrahmani, Ion Vasiloi, David Santer, Denis Berdajs, Friedrich S. Eckstein, Oliver Reuthebuch

**Affiliations:** 1 Department of Cardiac Surgery, University Hospital Basel, 4031 Basel, Switzerland; 2 Surgical Outcome Research Center Basel, University Hospital Basel, University Basel, 4031 Basel, Switzerland

**Keywords:** coronary artery bypass grafting, myocardial protection, minimal extracorporeal circulation system, perfusion, microplegia, myocardial infarction

## Abstract

Background: We aimed to analyse the performance of minimal invasive extracorporeal circulation (MiECC) concomitantly with Microplegia, in patients with recent myocardial infarction (MI) undergoing urgent coronary artery bypass grafting (CABG) surgery. Methods: We included patients with a recent MI (≤7 days) undergoing isolated CABG surgery using MiECC. The primary endpoint was a major cardiovascular or cerebrovascular event (MACCE). In a secondary analysis, we compared our institutional Microplegia concept with the use of a crystalloid single-shot cardioplegic solution. Results: In total, 139 patients (mean ± standard deviation (SD) age 66 ± 10 years) underwent urgent CABG surgery using Microplegia; 55% (*n* = 77) of the patients had an acute MI within 1–7 days preoperatively; 20% (*n* = 28) had an acute MI within 6–24 h; and 24% (*n* = 34) had an acute MI within <6 h preoperatively. The number of distal anastomoses was a geometric mean of 4 (95% confidence interval 3–4). The MACCE and in-hospital mortality were 7% (*n* = 10) and 1% (*n* = 2), respectively. The results were confirmed in a secondary analysis comparing Microplegia with crystalloid cardioplegic solution (*n* = 271). Conclusion: The use of MiECC with Microplegia in urgent CABG surgery is feasible and safe and provides a straight-forward intraoperative setting. Therefore, it can also be considered to retain the benefits of MiECC in urgent CABG surgery.

## 1. Introduction

Coronary artery bypass grafting (CABG) represents the therapy of choice for patients with complex coronary artery disease [1,2,3]. Despite recommendations for off-pump surgery for patients with significant atherosclerotic aortic disease and high-risk patients, the majority of CABG operations are performed using extracorporeal circulation (ECC), especially in the case of urgent CABG surgeries [3,4,5].

To further improve outcomes after CABG, various efforts have been made within recent decades to implement new strategies and technologies. Among them, a minimized and closed form of ECC, the minimal extracorporeal circulation system (MiECC) was introduced to maintain the advantages of the ECC but to reduce artificial surfaces and avoid blood–air contact [6].

Based on excellent short-, mid-, and long-term outcomes, the use of MiECC in CABG surgery should be considered over the standard ECC, to decrease blood loss and the need for transfusion as well as to increase the biocompatibility of ECC [6,7,8,9,10,11,12,13,14,15,16,17]. In addition to MiECC, at our institution we use the Myocardial Protection System (second-generation, MPS^®^) to deliver a refined Microplegia, which was shown to be superior regarding perioperative myocardial injury when compared to a single-shot crystalloid solution (Cardioplexol^®^ Bichsel, Interlaken, Switzerland) [7,8].

However, data analysing the use of MiECC in the setting of an urgent operation in patients with recent acute myocardial infarction (MI) is limited, even though this represents a crucial and, so far in studies, underrepresented patient population.

To address this major gap in clinical knowledge, we aimed to analyse our experience using MiECC in patients with recent MI undergoing urgent CABG surgery and in a secondary analysis to compare the use of our institutionally developed Microplegia concept with the use of a crystalloid single-shot cardioplegia.

## 2. Materials and Methods

### 2.1. Study Design

In our centre, standard perfusion strategies for isolated CABG surgery comprise on-pump surgery using MiECC or off-pump coronary artery bypass grafting (OPCABG). ECC is mostly applied in emergency situations with potential need for concomitant procedures such as beating heart surgery, valve surgery, or other non-CABG surgeries. Consequently, since ECC is only used in special situations and a minority of isolated CABG surgeries (*n* = 124), these patients were excluded and also not considered for comparison with MiECC.

To investigate the quality of MiECC in consecutive patients over the age of 18 undergoing urgent CABG in patients with recent MI, we only included patients with an MI within the last 7 days prior to surgery based on our own observational data and excluded all patients without prior MI or MI ≥ 8 days.

**Figure 1:**
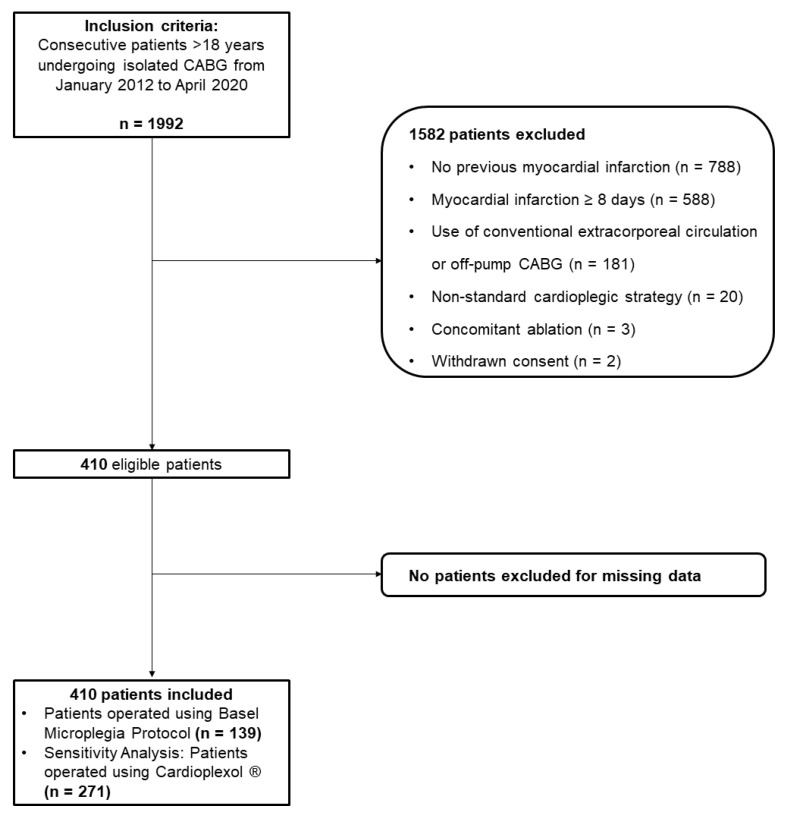
Flowchart; CABG denotes coronary artery bypass grafting.

For this analysis, we further excluded patients with OPCAB surgery, non-standard cardioplegic strategy, or concomitant ablation (Figure 1). Patients with concomitant ablation were excluded, since we aimed for a homogenous study cohort with a minimum of additional confounders.

To further generalize our findings, we performed an additional analysis comparing patients operated on using MiECC and Microplegia (current standard approach) with a historical cohort operated on using MiECC and a single-shot cardioplegia (further details are provided in the section “Cardioplegia strategy and technical details”).

Using a prospectively maintained institutional registry (Intellect 1.7, Dendrite Clinical Systems, Henley-on-Thames, UK), we identified all suitable patients after January 2012. This registry, where data have been regularly controlled for completeness and accuracy, was used to export the clinical data [7,8,15].

Furthermore, we assessed, as the primary outcome, MACCE defined as postoperative stroke, MI, or all-cause death before discharge. Stroke was defined as episode of neurological dysfunction caused by a focal cerebral infarction and typically confirmed with imaging. MI was diagnosed according to current clinical guideline practice [18].

Blood results such as high-sensitivity cardiac troponin T (hs-cTnT), creatine kinase (CK), and CK-myoglobin-binding (CK-MB) were measured according to the standard algorithm in our hospital, starting on postoperative day (POD) 1 and continuing on a daily basis until a decreasing trend was observed. However, since the preoperative dynamic of these biomarkers, which per se are elevated in patients with recent MI, could not be precisely implemented in a statistical model, we chose MACCE as the primary endpoint.

### 2.2. Cardioplegia Strategy and Technical Details

In our institution, we use a closed Mini-ECC-system type 1 with a priming volume of about 600 mL. The system is composed of a Bio-Console^®^ 560 (Medtronic Inc., Minneapolis, MN, USA), a Medtronic centrifugal pump, and an Affinity FUSION^®^ oxygenator (Medtronic Inc.). Targeted flow rates are 2 L/min/m^2^ [6].

**Figure 2:**
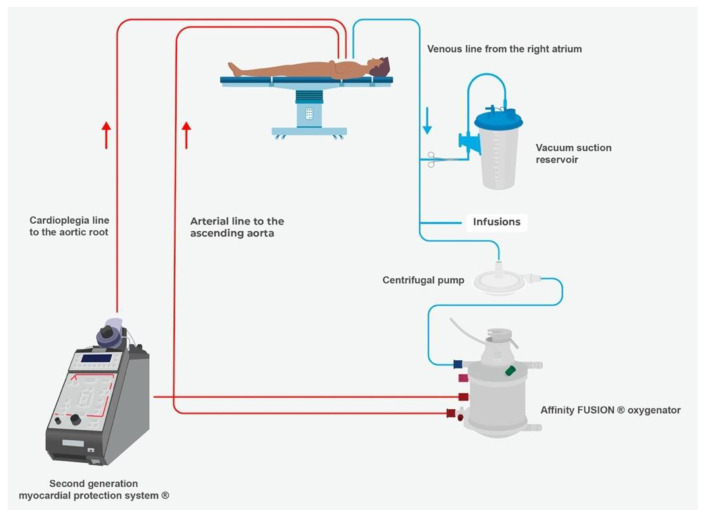
It shows the concept of the Myocardial Protection System (MPS^®^) as an additional tool to the minimal extracorporeal circulation (MIECC) system to apply Microplegia.

When using the MiECC system, only a few cardioplegic solutions are applicable. High-volume crystalloid cardioplegic solutions are not feasible due to the closed concept of MiECC leading to volume overload. In order to further improve our perfusion strategy when using MiECC, we added the second-generation MPS^®^ to apply an institutional, refined Microplegia (Basel Microplegia Protocol, BMP) in 2017 (Figure 2). 

High volumes of patient blood, gained from the oxygenator, enriched with potassium (20 mmol/L for the first two minutes and 13 mmol/L for the next two minutes), magnesium (1.6 g/L), and lidocaine (40 mg/L) (therefore, high-volume but euvolemic Microplegia) are applied with a targeted flow of 300 mL/min for 4 min during induction and 2 min in a 20 min interval for repetition. Before declamping, a hot shot (warm patient blood) is given for 1 min (without potassium). This concept was shown to be feasible, reliable, and beneficial compared to a single-shot, low-volume crystalloid cardioplegic solution (Cardioplexol^®^ Bichsel, Interlaken, Switzerland; composition: 10 mmol/100 mL potassium, 16.2 mmol/100 mL Magnesium; 1.1 mmol/100 mL procaine, 29.6 mmol/100 mL xylitol), regarding perioperative myocardial damage in patients without recent MI [7,8,15]. This single-shot cardioplegia, which is applied directly and manually via the aortic root, was used in our institution before introduction of the Microplegia. Since the Microplegia showed excellent results, the combination of MiECC and Microplegia became routine in isolated CABG in our institution [7,8,15]. Further details of our Microplegia solution, as well as the use of single-shot cardioplegia, were previously described in detail [7,8].

Surgical strategy remains similar when using Microplegia compared to the use of MiECC with single-shot cardioplegia, except for the induction phase of 4 min, repeated Microplegia administration at 20-min intervals as well as the “hot-shot” application prior to declamping [7,8]. In brief, after median sternotomy and heparinization, the ascending aorta and the right atrium were cannulated. While we aimed for 34 °C core temperature when introducing the Microplegia concept, we nowadays aim for a normothermic core temperature. Topical cooling of the heart was not performed as standard. Routine postoperative anticoagulation consisted of lifelong 100 mg aspirin daily and a second anti-platelet medication for 12 months after myocardial infarction.

### 2.3. Ethical Approval

The study was conducted according to the principles of the Declaration of Helsinki and was authorized by the ethical committee (EKNZ BASEC Req-2019-02383; ClinicalTrials.gov ID NCT04309994). Informed consent was waived by the ethical committee.

### 2.4. Statistical Analysis

We carried out two main analyses. We first described our Microplegia treatment group summarizing continuous variables as mean ± standard deviation (SD) if normally distributed or as geometric means with confidence intervals back-transformed from the logarithmic scale if skewed. Categorical data were reported as numbers with percentage. 

For the comparison of MiECC in conjunction with Microplegia and single-shot cardioplegia, we conducted an inverse probability of treatment weighting (IPTW) analysis to derive the average treatment effect based on logistic regression. We included gender, body mass index (BMI), preoperative stroke, preoperative renal failure, time since myocardial infarction (MI), hypertension, NYHA class 3 or 4, current smoking status, main stem disease, and preoperative atrial fibrillation as covariates into the propensity model. Time since MI was categorized into <6 h, 6–24 h, and 1–7 days. We selected these variables because they might be associated with the risk of MACCE, which is the primary outcome.

We censored treatment weights exceeding the 1st and 99th percentile [19] or 10, respectively, and calculated standardized differences for each variable to assess residual imbalances between the groups, using the formulae proposed by Austin et al. [20]. Differences between the treatment groups (Microplegia and single-shot cardioplegia) before and after IPTW were expressed as standardized differences to assess comparability, independently of the number of observations; the standardized differences are displayed in Supplemental Figure S1. We also calculated *p* values using linear regression for normally distributed continuous variables, linear regression on the log scale for skewed variables, and logistic regression for binary and multinomial logistic regression for categorical variables. Confidence intervals and *p*-values are two-sided; a *p*-value below 0.05 is considered significant. All analyses were performed by a biostatistician (BG) using Stata 16.0 (Stata Corp, College Station, TX, USA).

## 3. Results

### 3.1. Preoperative Data

From January 2012 until April 2020, 1992 patients underwent isolated CABG surgery. In total, 1582 patients were excluded, and the study cohort consisted of 139 patients operated on using Microplegia, while 271 patients were operated on using single-shot cardioplegia and are analysed within the secondary analysis (Figure 1).

Mean ± SD age was 66 ± 10 years, and the majority of patients were men (*n* = 122, 88%). Overall, 55% (*n* = 77) of the patients had an acute MI within 1–7 days preoperatively, whereas 20% (*n* = 28) had an acute MI within 6–24 h, and 24% (*n* = 34) had an MI < 6 h preoperatively. In order to derive unconfounded effect estimates, we conducted propensity modelling and calculated IPT-weighted effect sizes intended to express the influence of the cardioplegic approach, rather than reflecting patient characteristics. Preoperative data are presented in Table 1.

### 3.2. Intraoperative Data

A left internal mammary artery (LIMA) was used as the graft in 97% of the operations (*n* = 135). Total arterial revascularization was performed in 17% (*n* = 23), and the number of distal anastomoses were a geometric mean of 4 (95% CI 3–4). Mean ± SD procedure time (from skin to skin) and aortic cross-clamp time were 234 ± 64 min and 67 ± 20 min, respectively. Intraoperative data are provided in Table 2.

### 3.3. Postoperative Data

Inotropic support at the end of the operation was necessary in 67 patients (48%). Geometric mean time and CI on the intensive care unit (ICU) and length of hospital stay were 2 (2 to 2) and 9 (9 to 10) days, respectively. MACCE was seen in 10 patients (7%). In-hospital mortality was 1% (*n* = 2). After IPTW, Microplegia and MACCE did not show an association, OR was 0.69, and 95% CI was 0.31 to 1.55; *p* = 0.37. Postoperative results are provided in Table 3. Similar findings were seen for male and female patients (Supplemental Table S1).

### 3.4. Comparison of Microplegia and Single-Shot Cardioplegia

In total, 139 patients undergoing CABG surgery using Microplegia were compared with 271 patients in whom single-shot cardioplegia was used. After IPWT, there were no significant differences regarding the preoperative data such as age, gender, and the time since myocardial infarction. Kernel density distribution and preoperative data before and after IPTW are presented in Supplemental Figure S1 and Table 1.

While the use of LIMA as a bypass graft, total arterial revascularisation, and numbers of distal anastomoses were comparable between patients operated on using Microplegia and Cardioplexol^®^, the mean ± SD aortic cross-clamp time was significantly longer in patients operated on with Microplegia (67 ± 21 versus 57 ± 19 min, *p* < 0.001). Intraoperative data are provided in Table 2. No significant difference was seen in postoperative MACCE and in hospital mortality comparing Microplegia and single-shot cardioplegia (*n* = 10 (7%) versus *n* = 28 (10%); *p* = 0.37 and *n* = 1 (1%) versus *n* = 11 (4%), *p* = 0.072). Postoperative data are presented in Table 3.

## 4. Discussion

This single-centre study aimed to analyse and report the experience of using MiECC in patients with recent acute MI undergoing urgent CABG surgery. We report three major findings:

First, the use of MiECC with Microplegia in patients with recent acute MI allows for good intraoperative conditions, indicated by a high number of distal anastomoses in an appropriate aortic-cross clamp time. Second, MiECC in urgent CABG surgery is safe and feasible, with low in-hospital mortality and frequency of MACCE. Third, the comparison of MiECC in conjunction with Microplegia with a historical cohort using another cardioplegic strategy (single-shot cardioplegia instead of Microplegia) confirmed the promising intra- and postoperative results when using MiECC. The significantly longer aortic cross-clamp time in patients operated on using Microplegia compared to patients operated on with single-shot cardioplegia is due to the differences in the administration protocol between the two cardioplegic solutions. While the single-shot cardioplegia was mostly only applied once, the Microplegia is given for 4 min during induction and 2 min in a 20 min interval for repetition as well as 1 min before declamping, which explains the 10 min longer aortic cross-clamp time in the Microplegia group.

These findings broaden the evidence on the use of MiECC in CABG surgery [6,7,8,9,10,11,12,13,14,15,16]. Additionally, these results also support the use of MiECC in the vulnerable and, so far, in studies underrepresented cohort of urgent CABG surgeries in patients with recent acute MI.

However, analysing and interpreting the outcomes in patients with (recent) acute MI undergoing CABG surgery are difficult for several reasons: First, the patients with recent MI represent a heterogenous patient population comprising patients that are hemodynamically unstable or even in shock. This is shown by the wide range of EuroSCORE II data sets (0.58–45.21) in our study population. Obviously, this affects the outcome and is difficult to be encompassed within a study protocol. Second, established outcome parameters for perioperative myocardial damage reflecting the quality of cardiac protection (hs-cTn, CK, and CK-MB) are complicated to be interpreted, since they are by definition elevated in patients with MI. The dynamics of these biomarkers from preoperative MI until the early postoperative period are too heterogeneous to be used in a statistical model. We decided to include all patients with previous MI within 7 days, since these patients were excluded or are underrepresented in previous studies regarding the performance of MiECC in isolated CABG surgery [7,8,10,15]. Nevertheless, to overcome the above-mentioned issues, further randomized trials with high patient numbers are needed to extend the knowledge in this field and to analyse and compare various protection strategies.

There is still an ongoing discussion concerning the optimal perfusion strategy in urgent CABG surgeries. Gaudino et al. could show that, in patients with post-infarction cardiogenic shock, off-pump CABG and the use of cardiopulmonary bypass (CPB) did not differ regarding in-hospital outcome [21]. However, use of CPB and the LIMA at the time of the operation was beneficial regarding follow-up survival [21]. In total, 181 patients were excluded in our analysis due to the use of conventional ECC (*n* = 124) or OPCABG (*n* = 57) instead of MiECC. As conventional ECC is mostly applied in emergency operations with the potential need for concomitant procedures such as valve surgery, this might embed a certain selection bias [7,8,15]. Nevertheless, our data show that, in a centre with broad experience with MiECC, the use of MiECC in urgent CABG surgery is feasible and safe and, therefore, should be considered to retain the benefits of MiECC also in urgent CABG surgery.

Some limitations have to be considered when interpreting the above-mentioned findings. First, based on the novelty of the combined use of the MiECC and the MPS systems, this was an observational single-centre study, so, external validation of our findings is preferable. Second, due to the inclusion and exclusion criteria of this study, the study cohort is relatively small, so, the generalizability of our results may be challenged. Additional studies with larger patient cohorts are needed to further deepen the evidence of this topic. Third, due to the retrospective analysis and as mentioned above, the study design is susceptible to selection bias for the patients operated on using MiECC.

## 5. Conclusions

In a centre with broad experience with MiECC, the use of MiECC with Microplegia in urgent CABG surgery is feasible and safe and provides a straight-forward intraoperative setting. Therefore, it can be considered to also retain the benefits of MiECC in urgent CABG surgery.

## Figures and Tables

**Table 1:** Preoperative data.

	Before IPTW				After IPTW			
	Single-Shot Cardioplegia *n* = 271	Microplegia *n* = 139	Diff. ^‡^	*p*	Single-Shot Cardioplegia *n* = 271	Microplegia *n* = 139	Diff. ^‡^	*p*
Age, years, mean ± SD	66 ± 10	66 ± 10	−0.039	0.71	66 ± 10	66 ± 10	−0.043	0.68
Female gender, n (%)	48 (18%)	17 (12%)	0.154	0.15	43 (16%)	24 (17%)	−0.038	0.76
BMI, kg/m^2^, mean ± SD	27 ± 4	28 ± 4	0.052	0.62	27 ± 4	28 ± 5	0.046	0.66
Diabetes, n (%)	69 (25%)	44 (32%)	−0.137	0.18	68 (25%)	48 (35%)	−0.208	0.072
Current smoker, n (%)	73 (27%)	40 (29%)	−0.041	0.69	74 (27%)	38 (28%)	−0.006	0.96
Hypercholesteremia, n (%)	195 (72%)	95 (68%)	0.079	0.45	188 (69%)	99 (71%)	−0.039	0.74
Hypertension, n (%)	223 (82%)	106 (76%)	0.149	0.15	218 (80%)	112 (81%)	−0.016	0.88
COPD, n (%)	21 (8%)	12 (9%)	−0.032	0.76	21 (8%)	11 (8%)	0.006	0.96
Renal disease, n (%)	10 (4%)	5 (4%)	0.005	0.96	9 (3%)	4 (3%)	0.036	0.72
Preoperative stroke, n (%)	15 (6%)	13 (9%)	−0.146	0.15	22 (8%)	10 (8%)	0.016	0.88
Peripheral artery disease, n (%)	26 (10%)	12 (9%)	0.033	0.75	25 (9%)	9 (6%)	0.111	0.27
NYHA III or IV, n (%)	93 (34%)	20 (14%)	0.477	<0.001	75 (28%)	40 (28%)	−0.021	0.87
Preoperative atrial fibrillation, n (%)	11 (4%)	7 (5%)	−0.047	0.65	11 (4%)	5 (4%)	0.028	0.78
Recent myocardial infarction				0.050				0.83
1–7 days, n (%)	163 (60%)	77 (55%)	−0.096		161 (59%)	87 (63%)	0.068	
6–24 h, n (%)	68 (25%)	28 (20%)	−0.118		63 (23%)	30 (21%)	−0.047	
<6 h, n (%)	40 (15%)	34 (24%)	0.243		47 (17%)	22 (16%)	−0.037	
Main stem stenosis, n (%)	97 (36%)	36 (26%)	0.215	0.044	88 (33%)	46 (33%)	−0.018	0.88
Three-vessel coronary artery disease, n (%)	244 (90%)	122 (88%)	0.072	0.48	243 (90%)	123 (88%)	0.038	0.72
Ejection fraction, %, mean ± SD	52 ± 11	52 ± 11	−0.016	0.88	52 ± 11	52 ± 12	−0.008	0.94
EuroSCORE II, geom. Mean (95% CI)	2 (2 to 3)	3 (2 to 3)	0.429	0.75	2 (2 to 3)	2 (2 to 3)	0.425	0.65
Last preop. hs TnT *, ng/L, geom. Mean (95% CI)	158 (122 to 206)	142 (103 to 196)	0.113	0.20	160 (122 to 210)	135 (94 to 194)	0.091	0.059

^‡^ standardized difference expressing the difference independent of the number of observations; * max 48 h before surgery. COPD, chronic obstructive pulmonary disease. IPTW, inverse probability of treatment weighting. NYHA, New York Heart Association. SD, standard deviation. Note: Data are presented as mean ± standard deviation or geometric mean with 95% CI or as numbers (%).

**Table 2:** Intraoperative data.

	Before IPTW				After IPTW			
	Single-Shot Cardioplegia *n* = 271	Microplegia *n* = 139	Diff. ^‡^	*p*	Single-Shot Cardioplegia *n* = 271	Microplegia *n* = 139	Diff. ^‡^	*p*
Defibrillation, n (%)	41 (15%)	26 (19%)	−0.103	0.32	40 (15%)	25 (18%)	−0.096	0.38
Arteria Radialis, n (%)	62 (23%)	28 (20%)	0.067	0.53	62 (23%)	27 (20%)	0.079	0.48
Use of BIMA, n (%)	41 (15%)	21 (15%)	0.001	1.00	43 (16%)	20 (14%)	0.046	0.68
Use of RIMA, n (%)	43 (16%)	21 (15%)	0.021	0.84	46 (17%)	20 (14%)	0.069	0.54
Use of LIMA, n (%)	256 (94%)	135 (97%)	−0.133	0.23	255 (94%)	136 (98%)	−0.203	0.055
Total arterial revascularisation, n (%)	42 (15%)	23 (17%)	−0.029	0.78	42 (15%)	23 (16%)	−0.026	0.81
Number of distal anastomoses, geom. mean (95% CI)	4 (3 to 4)	4 (3 to 4)	0.747	0.92	4 (3 to 4)	4 (3 to 4)	0.743	0.71
Clamping time in min, mean ± SD	56 ± 18	67 ± 20	0.571	<0.001	57 ± 19	67 ± 21	0.531	<0.001
Perfusion time in min, mean ± SD	91 ± 27	100 ± 31	0.323	0.002	91 ± 28	99 ± 28	0.281	0.008
Duration of operation in min, mean ± SD	197 ± 52	234 ± 64	0.631	<0.001	197 ± 54	234 ± 63	0.635	<0.001

^‡^ standardized difference expressing the difference independent of the number of observations. BIMA, both internal mammary arteries. Diff., standardized differences to express the difference independent of the number of observations. LIMA, left internal mammary artery. RIMA, right internal mammary artery. SD, standard deviation. Note: Data are presented as mean and standard deviation or as numbers (%).

**Table 3:** Postoperative data.

	Before IPTW				After IPTW			
	Single-Shot Cardioplegia *n* = 271	Microplegia *n* = 139	Diff. ^‡^	*p*	Single-Shot Cardioplegia *n* = 271	Microplegia *n* = 139	Diff. ^‡^	*p*
Sepsis, n (%)	10 (4%)	0 (0%)	0.277	0.018	10 (4%)	0 (0%)	0.282	0.018
MACCE, n (%)	26 (10%)	10 (7%)	0.087	0.42	28 (10%)	10 (7%)	0.104	0.37
Pulmonary infection, n(%)	20 (7%)	11 (8%)	−0.02	0.85	21 (8%)	8 (6%)	0.084	0.40
Postoperative renal failure, n (%)	15 (6%)	5 (4%)	0.093	0.39	16 (6%)	5 (3%)	0.110	0.35
Sternal infection, n (%)	16 (6%)	3 (2%)	0.191	0.10	16 (6%)	2 (1%)	0.245	0.022
Atrial fibrillation at discharge, n (%)	68 (25%)	34 (24%)	0.015	0.89	67 (25%)	35 (25%)	−0.001	0.99
Postoperative Stroke, n (%)	13 (5%)	6 (4%)	0.023	0.83	15 (5%)	5 (4%)	0.079	0.47
Postoperative myocardial infarction, n (%)	10 (4%)	3 (2%)	0.091	0.41	10 (4%)	4 (3%)	0.044	0.72
Reoperation for bleeding, n (%)	16 (6%)	8 (6%)	0.006	0.95	15 (5%)	7 (5%)	0.024	0.82
Mechanical circulatory support *	17 (6%)	9 (6%)	−0.008	0.94	16 (6%)	6 (4%)	0.075	0.44
Intubation >72 h, n (%)	15 (6%)	0 (0%)	0.342	0.004	16 (6%)	0 (0%)	0.350	0.004
Operative mortality, n (%)	11 (4%)	2 (1%)	0.161	0.17	11 (4%)	1 (1%)	0.193	0.072
Stay on intensive care unit, days	3 (2 to 3)	2 (2 to 2)	0.278	0.013	3 (2 to 3)	2 (1 to 2)	0.245	0.006
hs-cTnT POD 1, ng/L	645 (556 to 749)	602 (484 to 748)	0.261	0.88	655 (556 to 772)	542 (442 to 663)	0.226	0.40
hs-TnT max, ng/L	725 (622 to 845)	689 (563 to 844)	0.273	0.99	738 (627 to 870)	628 (519 to 760)	0.241	0.35
CK-MB POD 1, ug/L	22 (20 to 25)	22 (19 to 25)	0.415	0.46	23 (20 to 25)	21 (18 to 23)	0.373	0.050
CK-MB max, ug/L	22 (20 to 25)	23 (20 to 27)	0.426	0.83	23 (20 to 26)	22 (19 to 26)	0.382	0.26
CK POD 1, U/L	643 (593 to 697)	674 (603 to 753)	0.536	0.68	645 (593 to 701)	642 (581 to 709)	0.519	0.51
CK max, U/L	842 (777 to 913)	826 (730 to 935)	0.483	0.54	845 (777 to 919)	785 (698 to 882)	0.461	0.90
Length of hospital stay, days	10 (10 to 11)	9 (9 to 10)	0.535	0.018	10 (10 to 11)	9 (9 to 10)	0.515	0.010

^‡^ standardized difference expressing the difference independent of the number of observations. CK, creatine kinase. CK-MB, creatine kinase MB. MACCE, major adverse cardiovascular or cerebrovascular events. Hs-cTnT, high-sensitivity cardiac troponin T. * defined as intra-aortic balloon pump, extracorporeal membrane oxygenator, or Impella.

## Data Availability

The data that support the findings of this study are available from the corresponding author, upon reasonable request.

## Supplementary Materials

The following supporting information can be downloaded at: https://www.mdpi.com/article/10.3390/jcm11247488/s1. Figure S1: It shows performance of the propensity score (PS) modelling. As can be seen in (a), distribution of PS in both treatment groups overlap almost completely with centers of mass close together. Part (b) shows that standardized differences between treatment groups are markedly below 0.1 with respect to all variables in the model, indicating good balance; Table S1: Outcome by gender.
