# Supplementary material for: Minimal Extracorporeal Circulation and Microplegia in the Setting of Urgent Coronary Artery Bypass Grafting
Source: J Clin Med. 2022 Dec 17;11(24):7488. doi: 10.3390/jcm11247488 (PMC9781499; doi:10.3390/jcm11247488)
Supplement: Supplementary file 1 [file jcm-11-07488-s001.zip › jcm-2006053-supplementary.pdf]

a)

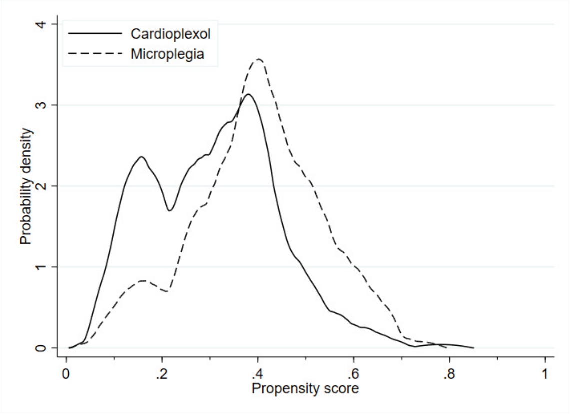

b)

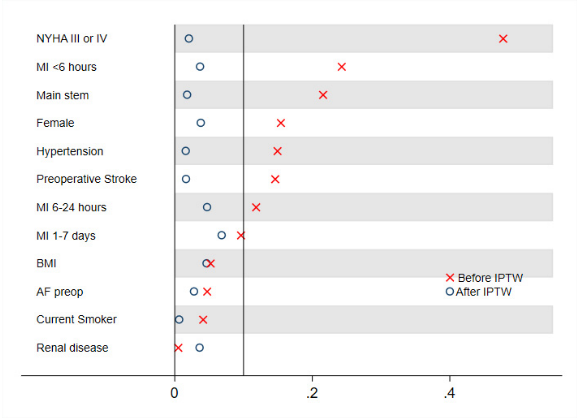

Supplemental Figure S1. shows performance of the propensity score (PS) modelling. As can be seen in a), distribution of PS in both treatment groups overlap almost completely with centers of mass close together. Part b) shows that standardized differences between treatment groups are markedly below 0.1 with respect to all variables in the model, indicating good balance.

Supplemental Table S1. Outcome by gender

	Before IPTW for Microplegia				After IPTW for Microplegia			
	Men, n = 345	Women, n = 65	SD	p	Men, n = 345	Women, n = 65	SD	p
Age	65 ± 10	68 ± 10	0.254	0.061	65 ± 11	68 ± 8	0.281	0.019
Euroscore2	2 (2 to 2)	4 (3 to 5)	0.679	0.016	2 (2 to 2)	4 (3 to 5)	0.651	0.015
Length of stay	10 (9 to 10)	11 (10 to 13)	0.671	0.618	10 (9 to 10)	11 (10 to 13)	0.679	0.459
ICU stay	2 (2 to 2)	3 (2 to 4)	0.502	0.143	2 (2 to 2)	3 (2 to 4)	0.432	0.072
Operative mortality	11 (3%)	2 (3%)	0.006	0.962	9 (3%)	1 (2%)	0.049	0.681
Intubation >72h	10 (3%)	5 (8%)	-0.215	0.069	8 (2%)	4 (6%)	-0.175	0.114
Postoperative MI	7 (2%)	6 (9%)	-0.316	0.006	8 (2%)	6 (9%)	-0.290	0.034
Postoperative Stroke	15 (4%)	4 (6%)	-0.081	0.527	16 (5%)	3 (5%)	-0.001	0.992
Postoperative renal failure	16 (5%)	4 (6%)	-0.067	0.604	15 (4%)	3 (5%)	-0.029	0.826
Pulmonary infection	24 (7%)	7 (11%)	-0.134	0.290	23 (7%)	5 (8%)	-0.045	0.718
MACCE	28 (8%)	8 (12%)	-0.139	0.277	28 (8%)	7 (11%)	-0.101	0.494
